# Severe Inhibition of Long-Chain Acyl-CoA Enoylhydratase (EC 4.2.1.74) in a Newborn Foal Suffering From Atypical Myopathy

**DOI:** 10.3389/fvets.2021.765623

**Published:** 2021-10-26

**Authors:** Johannes Sander, Michael Terhardt, Nils Janzen

**Affiliations:** ^1^Screening-Labor Hannover, Hanover, Germany; ^2^Hanover Medical School, Hanover, Germany

**Keywords:** long-chain enoyl-CoA hydratase (EC 4.2.1.74), vertical toxin transmission, hypoglycin A (HGA), methylenecyclopropylglycine (MCPG), atypical myopathy, newborn foal

## Abstract

In horses, congenital defects of energy production from long-chain fatty acids have not been described so far. In contrast, inhibition of fatty acid degradation caused by the toxins hypoglycin A and methylenecyclopropylglycine from various maple species are observed frequently. These non-proteinogenic aminoacids are passed on placentally to fetuses or with collostrum or milk to newborn foals. Nevertheless, newborn foals become very rarely symptomatic. Vertical transmission apparently is not sufficient to induce clinical disease without a particular genetic constellation being present. One of these rare cases was investigated here using samples from a mare and her foal. Intoxication by hypoglycin A and methylenecyclopropylglycine is also of interest to human pathology, because these toxins have caused fatal poisonings after consumption of certain fruits many times, especially in children. Maple toxins, their metabolites and some short-chain acyl compounds were quantified by ultrahigh-pressure liquid chromatography/tandem mass spectrometry. An comprehensive spectrum of long-chain acylcarnitines was prepared using electrospray ionization tandem mass spectrometry. Organic acids and acylglycines were determined by gas chromatography mass spectrometry. For evaluation, results of other horses poisoned by maple material as well as unaffected control animals were used. In the serum of the foal, hypoglycin A was detected at a low concentration only. Toxin metabolites reached <3.5% of the mean of a comparison group of horses suffering from atypical myopathy. The spectrum of acylcarnitines indicated enzyme inhibition in short-chain and medium-chain regions typical of acer poisoning, but the measured concentrations did not exceed those previously found in clinically healthy animals after maple consumption. The values were not sufficient to explain the clinical symptoms. In contrast, a remarkably strong enrichment of tetradecenoylcarnitine and hexadecenoylcarnitine was observed. This proves a blockade of the long-chain enoyl-CoA hydratase (EC 4.2.1.74). Vertical transfer of maple toxins to a newborn foal is sufficient for induction of clinical disease only if there is an additional specific reactivity to the active toxins. This was found here in an inhibition of long-chain enoyl-CoA hydratase. Isolated dysfunction of this enzyme has not yet been reported in any species. Further studies are necessary to prove a specific genetic defect.

## Introduction

Several thousands of severe, often letal cases of atypical myopathy (AM) caused by the ingestion of seeds and seedlings of some *Acer* species have been observed in horses and other equids, occurring in all age groups from the very young to the very old animals [for review see Votion et al. (1)]. Hypoglycin A (HGA) and methylenecyclopropylglycine (MCPG) are the constituents responsible for this. Newborn foals, however, that do not yet consume green forage themselves and therefore can receive *Acer* toxins prenatally only via the placenta or postnatally with the milk are apparently very rarely affected by AM.

MCPA-CoA and MCPF-CoA, the active toxins formed from HGA and MCPG, are known to interrupt the energy production from fatty acids and certain amino acids. They inhibit mitochondrial acyl-CoA dehydrogenases and enoyl-CoA hydratases involved in the ß-oxidation of fatty acids (2–4). In view of the high postpartum requirement for products of the ß-oxidation of fatty acids and amino acid catabolism, one might assume that newborn foals are particularly vulnerable to maple toxins. However, so far, only one case of AM in a foal has been described in the literature (5).

The maple toxins HGA and MCPG have the structure of amino acids. Thus, they can pass through the placenta and enter the fetus like other amino acids. They are also secreted into the milk (6–8). Transplacental transport and secretion of amino acids into the milk, on the other hand are not primarily achieved by passive diffusion but are the results of active transport. And, in order to trigger toxic effects, HGA, and MCPG must be converted into the active toxins in the foal's organism after vertical transfer.

Based on the above facts, it must be assumed that placental passage of the toxins into the fetal circulation as well as secretion into the milk are limited. Concentrations sufficient to affect metabolism will therefore not be reached frequently. Furthermore, there is the possibility that the activity of the enzymes necessary for metabolizing maple toxins to MCPA-CoA and MCPF-CoA, cytosolic aminotransferase, and mitochondrial branched-chain dehydrogenase, is comparatively low in the tissues at the end of the fetal period or in the early neonatal period. This would mean that only a small proportion of the toxic amino acids entering the newborn foal's organism would be converted into active toxins.

On the basis of these considerations, we hypothesize that the low concentrations of active maple toxins to be expected in a foal fed with collostrum or milk will only lead to acute disease if there is a special, possibly genetically determined sensitivity.

Test material received by our laboratory from a mare/foal pair suffering from AM allowed us to investigate some aspects of this hypothesis and to draw tentative conclusions.

## Materials and Methods

### Anamnestic Details

A male foal weighing 50 kg had been nursed by his dam (Iceland pony, 5 years old) until the 7th day of life in a pasture in the neighborhood of *Acer pseudoplatanus*. When depression, insecure gait, weakness, and finally recumbency were detected in the foal on the 7th day postpartum, in view of the fact that cases of AM had already been confirmed in this pasture, the owner expressed the suspicion that it could be AM. Dam and foal were seen by a veterinarian at the 8th day postpartum. At this time, the dam was described as being weak and stiff but she did not appear to be in acute distress. The foal on the other hand was moribund. Enzyme activities in serum were 45,305 U/L for creatine kinase (CK) in the foal and 19,515 U/L in the dam (ref. <200). Lactate dehydrogenase (LDH) activity in the serum was 2,254 U/L for the foal and 12,833 U/L for the dam (ref. <250). Levels of aspartate aminotransferase (AST) activity in the serum were also greatly elevated (foal 2,656 U/L, dam 16,999 U/L, ref. <50). High concentrations of myoglobin were detected in the urine of the foal (3,990 μg/L) and the dam (1,060 μg/L, ref. <25). The results obtained are compatible with the diagnosis of AM.

The dam recovered within 3 days on a maple free diet of hay and oats. The foal was euthanized because of the severity of symptoms and obviously poor chance of survival. The veterinarian collected samples for diagnostic purposes and requested the toxicological analyses described below.

### Materials and Data Available

- Foal: Serum, collected before euthanasia on the 8th day postpartum, urine collected immediately after death
- Dam: Serum and urine, collected on the 8th day postpartum
- Data for comparison were available from the diagnostic workup of our laboratory of 34 AM positive horses and 19 negative controls.

Data of all the horses were used for scientific purposes with owner-informed consent.

### Ultrahigh-Performance Liquid Chromatography/Tandem Mass Spectrometry (UPLC-MS/MS)

The quantification of HGA and MCPG plus their metabolites in serum and urine was carried out by UPLC-MS/MS as described in detail earlier (9–12). For all analyses, 25 μL of material were extracted with 300 μL of methanol containing the internal standards. Analysis was performed after butylation. For chromatographic separation 5 μL of the final extracts were injected onto an Acquity UPLC BEH C18 1.7 μm, 2.1 ×50 mm column (Waters). Gradient chromatography was performed using acetonitrile/water modified by 0.1% formic acid and 0.01% trifluoroacetic acid. Quantifications were done on a Xevo TQMS UPLC-MS/MS system (Waters, Eschborn, Germany), calculation of concentrations was conducted with single-point calibration. Concentrations of C4 to C10 acyl conjugates were also determined by this method in order to differentiate branched and unbranched C4 and C5 metabolites. The isomers of 2-MBC appeared in two separate peaks, the values of both isomers were added for quantification.

### Flow Injection-Tandem Mass Spectrometry (FI-MS/MS)

A comprehensive range of long-chain acylcarnitines (C12-C18) was quantified using a method originally developed for the study of infant blood (13, 14). This method has been adapted in our laboratory for the analysis of horse serum samples (15). For the study presented here, the serum was spotted onto filter paper (Ahlstrom-Munskjö, Germany). After air-drying, disks of 3.2 mm diameter were punched out (corresponding to 3.5 μl serum) and extracted with 100 μl methanol. After butylation with 50 μL HCl-butanol, drying and resolving the extracts were analyzed using flow injection tandem mass spectrometry without chromatographic separation. Analysis was done on a Waters TQD instrument (Waters, Eschborn, Germany) with an electrospray interface. The instrument was run in the ESI positive mode. Capillary voltage was 2.5 kV. Collision gas (argon) pressure was 2.4 ×10^−3^ mbar. The analysis was done as parent scan, the long chain hydroxylated AC, however, were measured in positive MRM mode because of their very low intensities. MS/MS results were calculated using the Neolynx software 4.1 (Waters), quantifications were done by comparing peak height to the respective d3- carnitine derivatives (C12-C18).

### Gas Chromatography Mass Spectrometry (GC-MS)

Organic acids and acylglycines were measured as commissioned service by a commercial human medical laboratory (MVZ Dr. Eberhard & Partner, Dortmund, Germany) who established a spectrum using gas chromatography mass spectrometry as described by Hoffmann et al. (16). The methods are used there as quality-controlled routine diagnostic procedures in human medicine.

## Results

### Toxins and Toxin Metabolites

**Figure 1:**
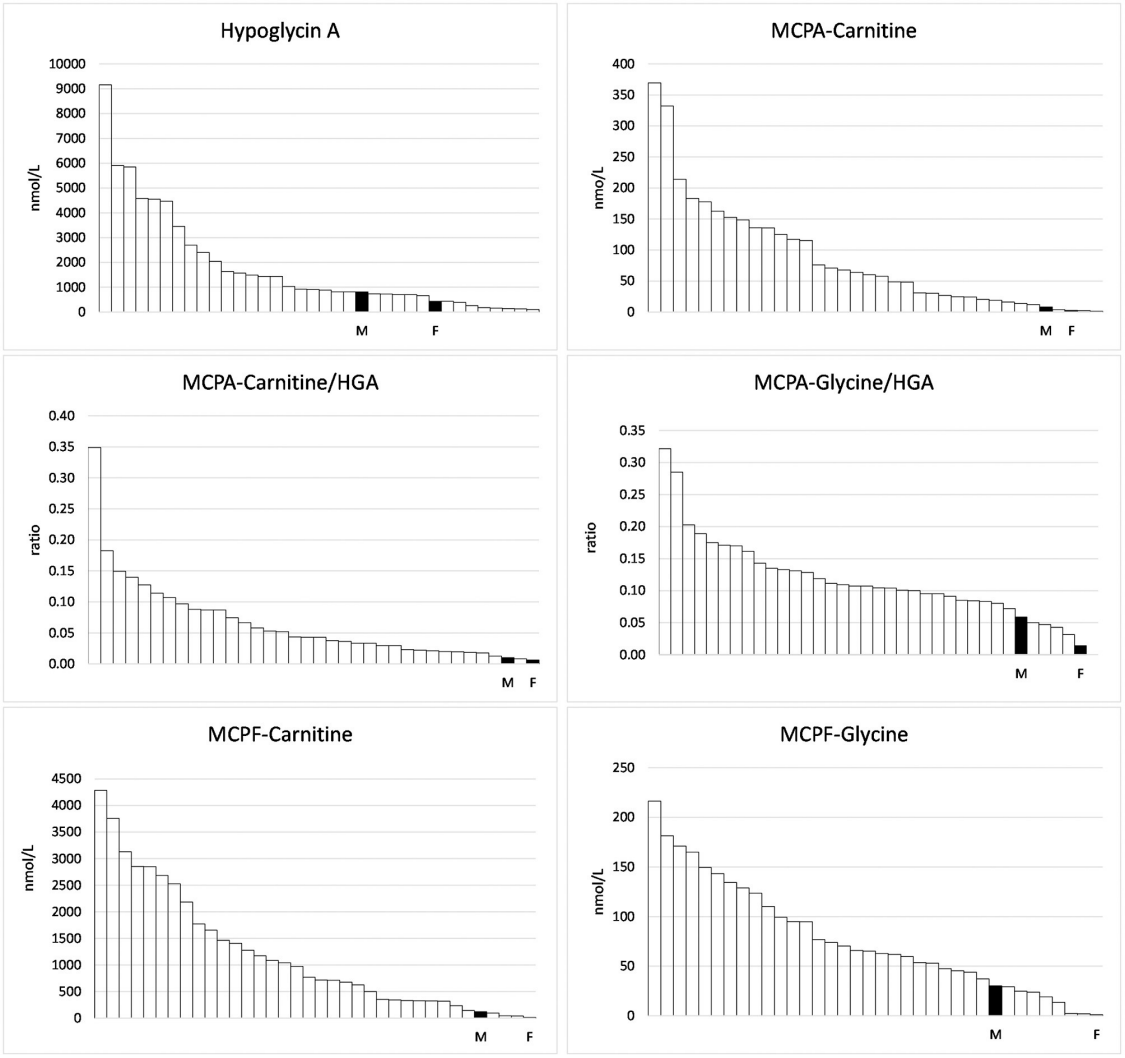
Hypoglycin A and toxin metabolites measured in the serum of 34 horses suffering from Atypical Myopathy plus the foal (F) and the mare (M).

Concentrations of HGA and toxin metabolites are shown in Table 1. In the serum of the foal, HGA, not present in body fluids of unaffected horses, reached only 53.3% of the level of the mare. However, the foal excreted unmetabolized HGA to a considerably higher extent than the mare. With 68 nmol HGA/mmol creatinine, the foal reached 125% of the mean of positive controls. Non-metabolized MCPG, which we had confirmed in other cases of maple poisoning of horses (17) was not detected in quantifiable concentrations. In serum, the metabolites MCPA-glycine and MCPA-carnitine derived from HGA as well as MCPF-carnitine and -glycine derived from MCPG, not detectable in unaffected horses, reached only a few percent of the mean of the group. This is clearly visible in Figure 1. The low levels corresponded to a very low excretion of toxin metabolites as compared to other AM horses (Table 1).

**Table 1:** Hypoglycin A and toxin metabolites in serum and urine of the foal and his mare.

	**Serum**				**Urine**			
	**Foal**		**Mare**		**Foal**		**Mare**	
	**nmol/L**	**% of mean** **of 34 AM horses**	**nmol/L**	**% of mean** **of 34 AM horses**	**nmol/mmol** **creatinine**	**% mean of** **14 AM horses**	**nmol/mmol** **creatinine**	**% mean of** **14 AM horses**
HGA	438	24.3	813	45.3	68	125	4.4	8.1
MCPA-G	6.2	3.3	48.0	25.3	969	8.4	2,550	22.2
MCPA-C	2.8	3.2	8.2	9.5	20.5	3.0	3.4	0.5
MCPF-G	1.2	1.6	30.2	39.2	57.0	1.8	1,567	49.3
MCPF-C	14.5	1.2	122	10.3	110	1.0	194	1.8

### Medium-Chain Acyl Carnitines in Serum and Urine

A high accumulation in the serum of the mare and the foal of butyrylcarnitine, a derivative of butyryl-CoA, which is metabolized by both medium-chain acyl-CoA dehydrogenase (MCAD, OMIM 607008) and short-chain acyl-CoA dehydrogenase (SCAD, OMIM 606885) was found (Table 2). The high concentrations indicate a pronounced inhibition of both enzymes especially in the foal. Increased concentrations in the serum of the foal and the mare alike of octanoylcarnitine and decenoylcarnitine are further indicators of inhibition of the MCAD enzyme. The branched-chain isobutyrylcarnitine, however, which indicates dysfunction of the isobutyryl-CoA dehydrogenase (OMIM 611283), was measured in increased concentrations in the serum of the foal only.

**Table 2:** Concentrations of acylcarnitines found in serum and urine of the foal and the mare.

**Acyl carnitines**	**Serum**					**Urine**				
	**Foal**		**Mare**			**Foal**		**Mare**		
	**μmol/L**	**% of mean** **of 34 AM horses**	**μmol/L**	**% of mean** **of 34 AM horses**	**Mean** **of 19 neg. controls**	**μmol/** **mmol crea**	**% mean of** **14 AM horses**	**μmol/** **mmol crea**	**% of mean** **of 14 AM horses**	**Mean** **of 11 neg.** **controls**
Butyryl	23	40.4	4.9	8.5	0.98	82.9	31.4	4.1	1.6	0.65
Hexanoyl	3.3	45.4	2.0	27.3	0.24	3.6	6.2	0.41	0.70	0.04
Octanoyl	0.4	32.4	0.37	30.0	0.02	1.0	13.7	0.17	2.22	0.01
Decenoyl	0.09	11.8	0.14	18.3	0.01	0.79	11.4	0.07	0.99	n.d.
Isobutyryl	8.3	129	1.3	19.8	1.5	138	181	7.4	9.7	2.3
Isovaleryl	7.0	42.8	1.1	6.5	0.31	41.8	64.8	2.7	4.2	0.28
Methylbutyryl	4.3	31.6	1.3	9.6	0.31	47.8	58.7	6.9	8.5	0.37

In the group of acylcarnitines with five carbon atoms high values for isovalerylcarnitine and 2-methylbutyrylcarnitine were measured especially in the serum of the foal indicating that he had a particularly pronounced inhibition of isovaleryl-CoA dehydrogenase (OMIM 607036) and 2-methylbutyryl-CoA dehydrogenase (OMIM 610006), which have functions in the degradation of leucine and isoleucine, respectively. Values for the unbranched valerylcarnitine were within the normal range in both animals.

### Long-Chain Acylcarnitines in the Serum

**Figure 2:**
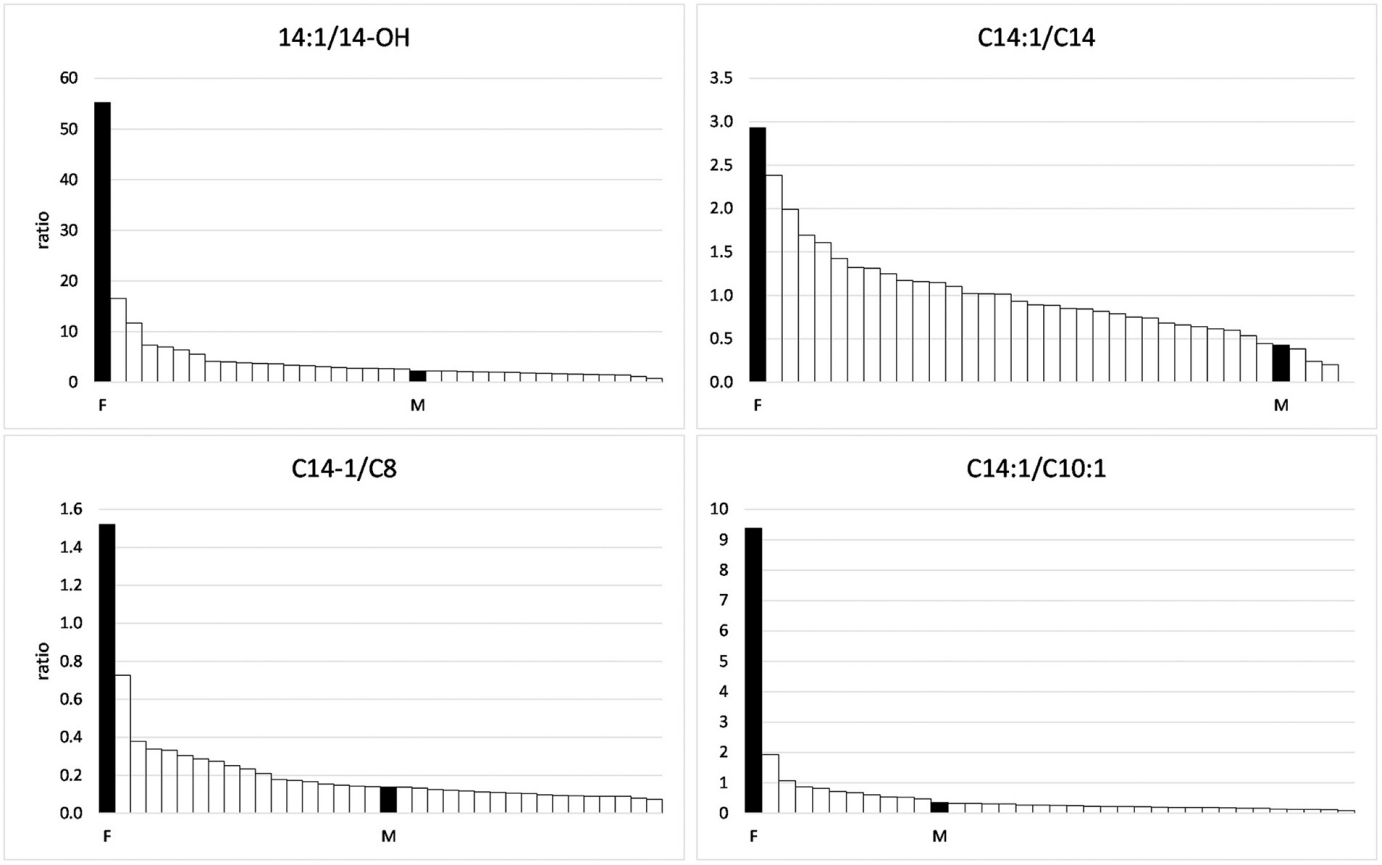
Ratios of C14:1 to other acylcarnitines in the group of horses suffering from Atypical Myopathy including the foal (F) and the mare (M).

In the range of long-chain acylcarnitines with chain lengths of 12–18 C-atoms, again, the values measured in the foal and the mare exceeded those of the negative controls (Table 3). This was true for both the saturated compounds that are products of the first step of the ß-oxidation spiral as well as for the products of the second step, the monounsaturated acylcarnitines. However, while values close to or below the mean of the comparison group were found for the saturated compounds, values for the unsaturated compounds C 14:1 and C16:1 were particularly high in the serum of the foal, amounting to 262 and 133% of the means of the comparison group, respectively. The particularly strong inhibition of the second reaction step of ß-oxidation in the foal becomes clear when the concentrations of C14:1 are put in relation to that of C14-OH as shown in Figure 2. While a ratio of 51.7/1 is calculated for the foal, the ratio for the mare is only 2.2/1.

**Table 3:** Long-chain acylcarnitines, measured by flow-injection MS/MS.

	**21 neg. controls**	**Foal**		**Mare**	
**Acyl-Carnitines**	**Mean** **nmol/L**	**Level** **nmol/L**	**% of mean** **of 34 AM horses**	**Level** **nmol/L**	**% of mean** **of 34 AM horses**
Dodecanoyl, C12	99.8	114	86.9	132	101
Dodecenoyl, C12:1	15.7	75	93.5	14	175
Myristoyl, C 14	27.6	52.8	103	30.4	59.6
Hydroxymyristoyl, C14-OH	4.7	2.8	17.0	5.8	35.2
Tetradecenoyl C14:1	14.1	155	262	13	22.1
Tertradecadienoyl C14:2	6.2	16	111	6.4	44.6
Palmitoyl, C 16	43.3	61.9	33.5	42.4	22.9
Hydroxypalmitoyl, C 16-OH	3.6	4.3	23.0	4.9	26.1
Palmitoleoy, C 16:1	17.7	103	133	12	15.4
Hydroxypalmitoleyl, C 16:1-OH	5.7	5.4	23.4	5.2	22.5
Stearyl, C 18	24.0	34.8	49.5	23.7	33.8
Hydroxy-octadecenoyl, C 18-OH	4.4	11.3	164	9	130
Oleoyl, C 18:1	37.1	68.5	42.0	31.7	19.4
Hydroxyoleyl, C 18:1-OH	4.4	6.9	41.3	6.4	38.3
Linoleyl, C 18:2	10.2	7.3	19.3	11.5	30.4
C 18:2-OH	6.5	10	64.3	17	109

### Organic Acids and Acylglycines in Urine

The analysis of the urine for organic acids provided results that confirmed and expanded those obtained by serum samples. In their entirety, they are a sign for a massively disturbed energy production from fatty acids and amino acids, a fact that is also underlined by the extremely high excretion of lactate in the foal. The results of the urine tests not only confirmed the impairment of the dehydrogenases and enoylhydratases shown for serum but also revealed various secondary metabolic disorders e.g., the accumulation of pyroglutamic acid as a sign of malfunction of the glutamyl cycle (Table 4).

**Table 4:** Excretion of organic acids in urine showing organaciduria which is severe in the foal, less pronounced in the mare, values in mg/g creatinine.

**Acid**	**Foal**	**Mare**	**Control**
lactic	3,800	17.5	4.9
glycolic	41	11.6	5.6
3-hydroxypropionic	1.8	0	0.6
3-hydroxybutyric	128	18	1.6
3-hydroxyisovaleric	66	1.2	0.3
2-methyl-3-hydroxybutyric	9.5	14.5	10.8
3-hydroxy-isovaleric	6.4	8.4	7.5
metylmalonic	0.4	0.9	1.6
2-ethyl-3-hydroxypropionic	33	41	1.4
4-Hydroxybutyric	7.8	2	0.5
ethylmalonic	47	36	1
methylsuccinic	33	30	7.6
glyceric	53	2.8	2.2
fumaric	2.5	0.5	0.6
mevalonic	0	0	1
glutaric	13.5	2	0.3
3-methylglutaric	0	0	0.2
3-methylglutaconic	1.6	3	0
adipic	106	9.7	0.4
pyroglutamic	131	8.6	17
2-hydroxy glutaric	114	31	3.4
3-hydroxy glutaric	6.2	3.4	1.6
3-hydroxy-3-methylglutaric	3.1	0	4.8
2-ketoglutaric	4.6	3.2	0
N-acetylaspartic	2.1	4.4	0.2
suberic	76	4.7	1
sebacic	0	1.3	0.4

While inhibition of the SCAD enzyme (OMIM 201470) in the mare as well as in the foal is indicated by high values obtained for ethylmalonic acid and 2-ethyl-3-hydroxypropionic acid, inhibition of the short/branched-chain acyl-CoA dehydrogenase enzyme (OMIM 600301), also called 2-methylbutyryl-CoA dehydrogenase, is proven by elevated excretion of 2-ethylhydracrylic acid (2-ethyl-3-hydroxypropionic acid) (18, 19). The pronounced excretion of the saturated dicarboxylic acids adipic and suberic acids and the ketone body 3-hydroxybutyric acid are clear indicators for the inhibition of the MCAD enzyme. Metabolites such as methylsuccinic acid or glutaric acid are typical markers of disorders in the metabolism of various amino acids.

Metabolic inhibition also led, as expected, to a greatly increased excretion of the corresponding acylglycines. The excretion of the acylglycines exceeded that of the corresponding carnitine compounds by two to three powers of ten. As a consequence of a nutrition from vegetable food, a large excretion of hippuric acid and phenylpropionyglycine (Table 5) was observed in the dam but not in the foal who did not eat plant material.

**Table 5:** Renal excretion of acylglycines and hippuric acid.

**Excretion^a^**	**Foal**	**Dam**	**Control**
Isovalerylglycine	25.7	40.6	0.3
Hexanoylglycine	23.6	29.5	0.1
Methylcrotonylglycine	2.1	8.3	0.1
Tiglylglycine	0.2	1.2	<0.1
Phenylpropionylglycine	0.5	15	<0.1
Hippuric acid	2.8	44.3	0.5

a *Excretion measured in μmol/mmol creatinine*.

## Discussion

The foal developed the full clinical picture of AM. The diagnosis is supported by strongly elevated values for muscle enzymes such as CK, LDH and by the excretion of myoglobin. Nevertheless, the biochemical findings suggest marked differences from other cases of AM. First, the very low serum level of HGA measured at the peak of clinical disease is noteworthy. It reached only 24.3% of the mean of the values found in the horses with AM used for comparison. The low level of HGA is further demonstrated by comparison with samples obtained from clinically healthy individuals. In a previous study (19), we found HGA levels that were higher than in the foal in 9 out of 12 clinically unsuspicious horses who had shared pasture with horses suffering from AM. A mean HGA level of 771 nmol (range: 268–2,327) was found in this group. The value of 438 nmol/L measured in the foal examined here represents only 56.8% of this mean. This corresponds in magnitude to values in 2 clinically healthy co-grazing horses observed by Baise et al. (20). Also noteworthy are the very low values found for MCPA and MCPF conjugates, as shown in Table 1. The concentrations of MCPA carnitine and MCPA glycine were only 0.63 and 1.4% of those of the parent compound HGA.

The fact that the foal developed the characteristic picture of AM despite only small amounts of vertically transferred toxins suggests a special readiness to react or, better, a special vulnerability of the biochemical processes of cellular energy production. An indication of a special point of attack comes from the analysis of the acylcarnitine concentrations. Very striking is the strong accumulation of the unsaturated long-chain acylcarnitines, especially of C14:1, the concentration of which was higher than in all compared AM horses. In contrast, the backlog of fatty acid degradation in the medium chain length region, although evident, was not very pronounced as compared to other AM horses. Hexanoyl-, octanoyl- and decenoylcarnitines reached only 45.4, 32.4, and 11.8% of the mean of the positive cases.

The strong accumulation of tetradecenoyl and hexadecenoyl carnitines makes it evident that the ß-oxidation of the long-chain fatty acids was blocked at the stage of hydration to the C14-OH and C16-OH compounds. This indicates an extensive loss of function of the enzyme responsible for this hydration step, long-chain enoyl-CoA hydratase (OMIM 609015, EC 4.2.1.74). The enzyme is, at least in humans, integrated into the mitochondrial trifunctional protein which also harbors the ß-hydroxy-acyl-CoA dehydrogenase and long-chain thiolase. However, there was no indication that these activities were negatively affected neither in the mare nor the foal.

An isolated defect of the long-chain ECH as a congenital disease has not yet been described for any species. Whether a genetic defect could underlie the present case would have had to be proven by appropriate genetic studies. Unfortunately, no suitable tissue was available. Thus, it must be left to the investigation of further cases to confirm this speculation.

In horses, congenital defects of the trifunctional protein have not been described yet, in humans they are rare (21). In newborn screening, an incidence of about 1:100 000 was found (22). As a differential diagnosis, a defect in very long-chain acyl-CoA dehydrogenase (OMIM #201475, EC 1.3.99.13) must also be considered. This disorder leads to an accumulation of C14:1 and other long-chain acylcarnitines. A genetic defect of the enzyme is known for humans [for review see van Calcar et al. (23)] and for dogs (24). However, this has not yet been observed in horses.

A risk of vertically transmitted HGA or MCPG might also exist for newborn babies because consumption of HGA and MCPG-containing fruits is widespread and increasingly popular. Based on the observations made here in a foal, one would have to conclude that newborns with a congenital defect of an enzyme of the ß-oxidation of fatty acids would be particularly at hazard. Further studies are needed to clarify a possible synergism of *Sapindaceae* toxins and special congenital defects.

## Data Availability Statement

The raw data supporting the conclusions of this article will be made available by the authors, without undue reservation.

## Author Contributions

JS was the principal investigator and conducted the conceptualization and experimental design. MT performed the analyses and was responsible for quality control. NJ supervised the interpretation of the study results and reviewed the draft. All authors contributed to the article and approved the submitted version.

## Conflict of Interest

The authors declare that the research was conducted in the absence of any commercial or financial relationships that could be construed as a potential conflict of interest.

## Publisher's Note

All claims expressed in this article are solely those of the authors and do not necessarily represent those of their affiliated organizations, or those of the publisher, the editors and the reviewers. Any product that may be evaluated in this article, or claim that may be made by its manufacturer, is not guaranteed or endorsed by the publisher.

## Abbreviations

AM: atypical myopathy
ECH: enoyl-CoA hydratase
Fi-MS/MS: flow injection-tandem mass spectrometry
HGA: hypoglycin A
MCPA: methylenecyclopropyl acetic acid
MCPF: methylenecyclopropyl formic acid
UPLC-MS/MS: ultra high pressure liquid chromatography-tandem mass spectrometry.
